# A new approach for the detection of genetic alterations utilizing modified loop-mediated isothermal amplification reaction (LAMP)

**DOI:** 10.1038/s41598-025-93086-2

**Published:** 2025-03-08

**Authors:** Aneta Cierzniak, Małgorzata Małodobra-Mazur, Miron Tokarski

**Affiliations:** 1 Genomtec SA, Ul. Bierutowska 57-59, 51-317 Wrocław, Poland; 2 Department of Forensic Medicine, Division of Molecular Techniques, Wroclaw Medical University, Skłodowskiej-Curie 52, 50-369 Wrocław, Poland

**Keywords:** Non-small-cell lung cancer, Diagnostic markers, Genetic testing, Laboratory techniques and procedures, Genetic techniques, Lab-on-a-chip

## Abstract

The increasing use of genetic testing for personalised therapy, highlights the need for rapid, reliable diagnostics. Current methods are hindered by complex workflows, requiring advanced equipment, skilled personnel, and invasive tissue sampling. Loop-mediated isothermal amplification (LAMP) has emerged as a more efficient alternative to traditional PCR. LAMP eliminates thermal cycling, allowing faster, more cost-effective tests, and is less sensitive to inhibitors, enabling testing from minimally processed samples. Although LAMP is newer and has a longer assay development time than PCR, its potential in oncology, particularly for detecting genetic changes, is promising. We have developed a LAMP-based method for detecting genetic variations, optimized for point-of-care testing. This technique uses modified primers with alterations at the 3’ end of either F2 or B2 primers, ensuring specificity for altered sequences. The assay only produces a positive signal when the genetic variant is present, distinguishing it from wild-type DNA. Our findings demonstrate that this method has high specificity and sensitivity, even in samples with both wild-type and mutated material. Paired with a portable device, this LAMP-based diagnostic method could revolutionize genetic alteration detection, offering quicker results and improving treatment outcomes, particularly for targeted therapies.

**Supplementary Information:**

The online version contains supplementary material available at 10.1038/s41598-025-93086-2.

## Introduction

Genetic variations are defined as heritable characteristics that are present in the population and play a crucial role in adapting to changing environmental conditions within the period of time. Throughout the years, many genetic testing technologies have allowed for detection and distribution evaluation of numerous genetic variations such as single nucleotide polymorphisms (SNPs), insertion or deletion (indels) of one or numerous nucleotides, copy number variations (CNVs) as well as chromosomal alterations, and other related to aberrations of substantial amount of genetic material^1^. Rapid diagnosis of genetic variations is particularly important in oncology. Nowadays, it is well known that cancers are the result of accumulation of mutations, especially in genes critical to tumorigenesis. Genetic mutations have been proven to be a molecular cause of numerous cancers. Although the cause of genetic mutation generation might vary depending on type of cancer, oncogenic variations are the main cause of cancer development and progression^2^. Precision oncology has been a growing field as research community pursue to better understand the specific pathophysiology of different types of cancer to reduce off-target side effects. Thus, it is important to identify the unique biomarkers of cancer patients that may predict their response to treatment. One of the cancers for which early molecular diagnosis is of paramount importance in the choice of treatment, while increasing the chances of survival, is non-small-cell lung cancer (NSCLC). It accounts for 80–85% of all lung cancers and has a relatively poor prognosis. In recent years, molecular targeted therapies have been developed that are safer and more effective for patients compared to classical chemotherapy, but required pretreatment detection of mutations. Molecular diagnostics is primarily based on the detection of mutations in *EGFR* (Epidermal Growth Factor Receptor) gene, where mutations are found in 10–15% of cases. Patients with confirmed alterations in the *EGFR* gene are treated with a group of drugs called tyrosine kinase inhibitors (TKIs), which reversibly inhibit the tyrosine activity thus inhibiting the proliferation of cancer cells^3,4^. In 2020, a large-scale study of patients newly diagnosed with advanced NSCLC in the US identified that ~ 23% of patients did not undergo genomic testing for any of the recommended therapeutic targets (*ALK*, *BRAF*, *EGFR*, and *ROS1* alterations) before first line treatment^5^. Often only NSCLC patients with the most advanced stage of disease (~ 65%) have the potential to receive treatment that specifically targets their genomic alterations^6^. This lack of proper diagnosis is largely a consequence of sophisticated workflows required by currently utilised diagnostic techniques such as next generation sequencing, Sanger sequencing, and multiplex Real-Time PCR that also increase testing cost^7–11^. These techniques require state-of the art laboratory equipment, sophisticated software, and highly trained laboratory personnel, together with invasive tissue sample collection. In addition, the multi-step nature of each of these diagnostic methods makes it difficult to adopt fully automated systems, not to mention rapid point-of-care testing (POCT) equipment. Those aspects, together with high cost of these methods, limit the access to recommended diagnostics for vast majority of patients.

Isothermal nucleic acid amplification techniques are a group of methods that, unlike classical PCR, only require one stable temperature throughout the amplification stage and as such has been developing rapidly in recent years. There are numerous modifications and technological differences of DNA/RNA amplification depending on the type of method. However, what is unique is the stable temperature of the whole process resulting in short turnaround time, sometimes within less than 10 min, whilst maintaining a high amplification efficiency^12^. What is more, these techniques are more resistant to numerous inhibitors, thus minimally processed or even crude samples can be used directly for nucleic acid amplification^13^. Of all isothermal techniques, Loop-mediated isothermal amplification (LAMP) is the most common method used for genotyping^12,14^. This method has evolved to detect single nucleotide variations as well as single or multiple nucleotide insertions or deletions. Incorporation of various types of modifications have made it possible to achieve a high power of discrimination between wild type and mutated genetic variants. The most common modification of the LAMP process is probe-based LAMP, where fluorescent probes are incorporated allowing for discrimination between genetic variant and wild-type sequence^15^. However, some of these approaches can make the reaction set-up more complex and, in several instances, require sophisticated calculations to subtract background signal^16^. Usefully, unlike PCR, LAMP can be directly used for detecting genetic variants in both DNA and RNA, with the latter being useful in haematological malignancies^17^. As LAMP is a single-step reaction with low equipment requirements, it can be used in POCT devices. In the case of NSCLC, this would not only facilitate rapid detection in the doctor’s office, but also speed up the process of targeted therapy selection and delivery, increasing the chances of patient survival^18^. LAMP is also advantageous for rapid genetic variation detection in cancer due to its short reaction time compared to other approaches and versatility in amplicon detection methods. Time to result including detection, can be shortened to less than one hour^19^. The most commonly used amplicon detection technology in LAMP-based amplification utilises florescence signal generated by intercalating dyes. End point product detection in LAMP can be even simpler by utilizing technology such as turbidimetric or colorimetric measurements, further enhancing the technique’s compatibility with POCT^20,21^. Similar to Real-Time PCR, utilisation of fluorescent dyes in the LAMP detection method greatly enhances the assay’s sensitivity, while also enabling melting-analysis^22^. With high amplification efficiency, like PCR, LAMP assays can achieve detection limits of few copies of genetic material per reaction, while also enabling quantitative measurements^19^.

Considering the advantages of LAMP technology outlined above, we have created a new approach for the detection of genetic variations utilising LAMP technology ideal for the point-of-care testing. Primers have been modified at the 3’end of either the F2 or B2 primers so they are specific to only the mutated sequence, therefore the amplification product is generated when mutated genetic variant is present and do not give positive signal with wild-type genomic DNA. Our results have shown that our assay dedicated to genetic variants in *EGFR* gene, is highly specific and sensitive, allowing for variant detection in a mixed samples of wild-type and mutated material without the added complexity and cost of probe-based approaches. Our detection method together with the portable device has the power to streamline and revolutionise the diagnosis of genetic alterations. This combination has the potential to lead to better treatment outcomes for not only in oncology but beyond into the ever-expanding field of targeted therapies.

## Materials and methods

### Genetic materials characterization

Synthetic gene fragments in the form of double stranded gBlocks® Gene Fragments (Integrated DNA Technologies, Iowa, U.S.), were used as templates modified to represent the human *EGFR* gene exhibiting a specific mutation. Detailed sequences are provided in the supplementary information.

#### gBlocks® used in reactions

- gBlock® 1. Represents a fragment of the human *EGFR* gene with NM_005228.5(EGFR):c.2235_2249del (p.Glu746_Ala750del) mutation in exon 19 (ClinVar RCV000150617.4).
- gBlock® 2. Represents a fragment of the human *EGFR* gene with NM_005228.5(EGFR):c.2240_2254del (p.Leu747_Thr751del) or NM_005228.3:c.2239_2253del15 or NM_005228.3:c.2238_2252del15 mutations in exon 19 (ClinVar RCV000154236.4).
- gBlock® 3. Represents a fragment of the human *EGFR* gene with NM_005228.5(EGFR):c.2230_2249delinsGTCAA (p.Ile744_Ala750delinsValLys) mutation in exon 19 (ClinVar RCV000150616.4).
- Additionally modified gBlock® 1. Representing a fragment of the human *EGFR* gene with NM_005228.5(EGFR):c.2235_2249del (p.Glu746_Ala750del) mutation present in such a manner that 10 nucleotides were inserted into the B2 primer binding site

Wild-type human DNA used as a background and negative control was isolated from blood collected from healthy donor. DNA was isolated using Invisorb® Spin Universal Kit (Invitek, Germany, catalogue number 1050100300) according to manufacturer protocol.

### Primers design

The F2 primers were modified so that 2 to 7 nucleotides at their 3’-end are identical to the nucleic acid sequence downstream of the fragment containing the genetic variant to be detected. Therefore, the F2 portion of FIP primer (forward inner primer) matches the nucleic acid sequence not only over the length of the F2 primer, but also the sequence after the insertion and/or deletion has occurred. The F2 primer is connected via its 5’-end, preferably via a TTTT bridge, to the 3’-end of the F1c primer, designed to be reverse and complementary to the nucleic acid fragment to be amplified (the 5’.

**Fig. 1:**
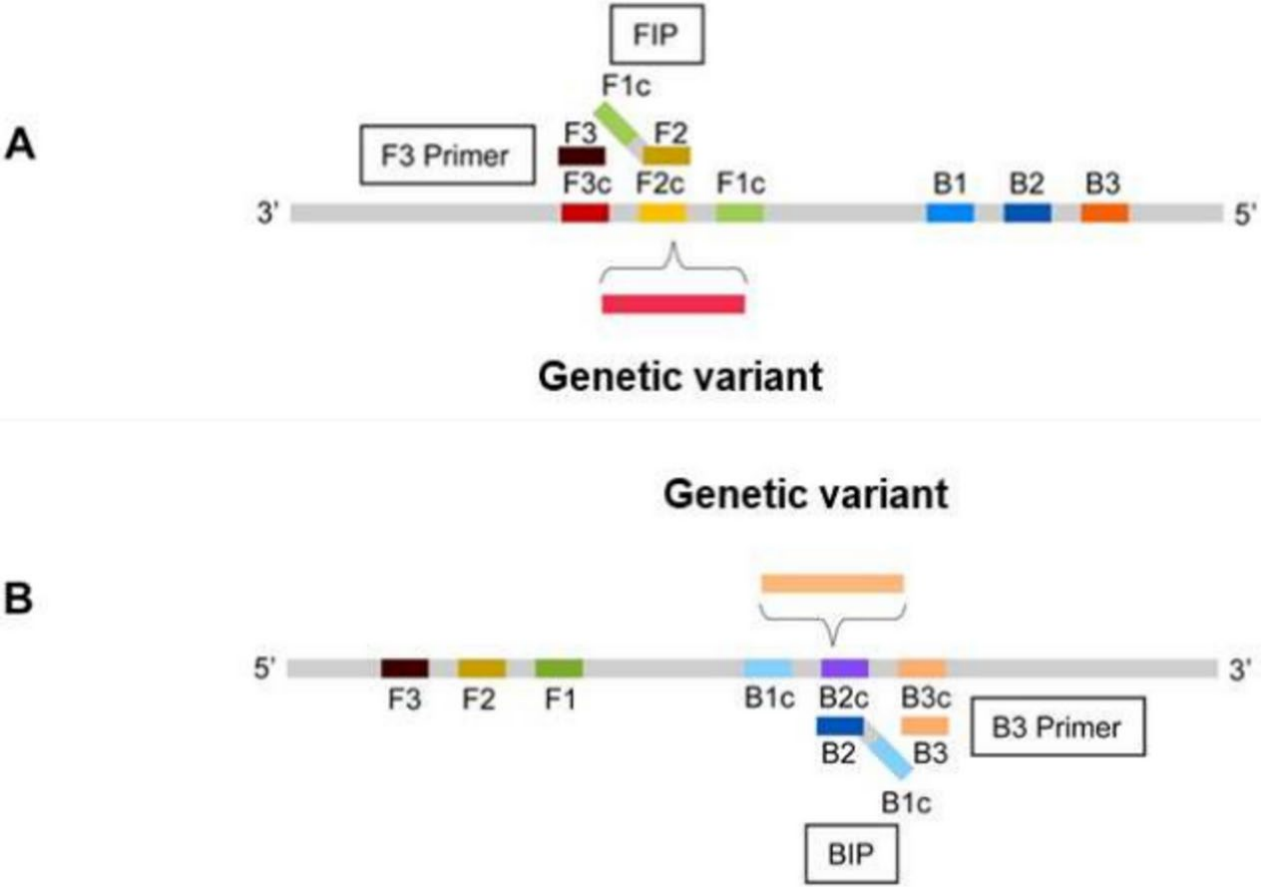
The principle of designing a FIP (**A**) or BIP (**B**) primer according to newly developed method.

 > 3’ strand), and located between the 3’-end of the F2 primer and the 5’-end of the B1c primer. At the same time remaining LAMP reaction primers, i.e. F3, B3, BIP (backward inner primer) and optional LF and/or LB loop primers, are designed as recommended in the literature, according to typical LAMP workflow, or using bioinformatics software such as PrimerExplorer (Eiken)^23,24^. The described modified method can also be applied to the B2 primer, in which case the BIP primer will be responsible for capturing the mutation. As shown on Fig. 1, the method develop by us relies on modifications of 2 to 7 nucleotide long portions of F2 or B2 fragments of BIP and FIP primers respectively.

The primer modifications made mean that as the F2 primers are specific only to mutated sequence, the amplification product is only generated when mutated genetic variant is present and do not give a positive signal in the presence of wild-type genomic DNA. In the reaction mixture, up to 3 different FIP primers can be used, whose F2 fragments are identical to the nucleotide sequences of 5 different deletions within *EGFR* gene:

- FIP1 designed to detect: NM_005228.5(EGFR):c.2235_2249del (p.Glu746_Ala750del)
- FIP2 designed to detect: NM_005228.5(EGFR):c.2240_2254del (p.Leu747_Thr751del) as well as NM_005228.3:c.2239_2253del15 and NM_005228.3:c.2238_2252del15
- FIP3 designed to detect: NM_005228.5(EGFR):c.2230_2249delinsGTCAA (p.Ile744_Ala750delinsValLys)

Primers’ sequences are presented in Table 1.

**Table 1:** The sequences of the primers used in the Real-Time LAMP reactions.

Primer	Sequence
F3	TGGATCCCAGAAGGTGAGAA
B3	GGGGTGGATACCAGCATG
FIP1	GACCCCCACACAGCAAAGCA-TCCCGTCGCTATCAAAACAT
FIP2	GACCCCCACACAGCAAAGCA-TCCCGTCGCTATCAAGGAATCTC
FIP3	GACCCCCACACAGCAAAGCA-TCCCGTCGCTGTCAAAACAT
BIP	GCTGCTCTGCTCTAGACCCT-AGAGGCCAGTGCTGTCT
LF	TCACATCGAGGATTTCCTTGTTG
LB	GCTCTAGTGGGTATAACTCCCTC
FIP—1 nucleotide modification	GACCCCCACACAGCAAAGCATTTTAAATTCCCGTCGCTATCAAA
FIP—2 nucleotide modification	GACCCCCACACAGCAAAGCATTTTAATTCCCGTCGCTATCAAAA
FIP – 3 nucleotide modification	GACCCCCACACAGCAAAGCATTTTATTCCCGTCGCTATCAAAAC
FIP—4 nucleotide modification	GACCCCCACACAGCAAAGCATTTTTTCCCGTCGCTATCAAAACA
FIP—6 nucleotide modification	GACCCCCACACAGCAAAGCATTTTCCCGTCGCTATCAAAACATC
FIP—7 nucleotide modification	GACCCCCACACAGCAAAGCATTTTCCGTCGCTATCAAAACATCT

### Real-Time LAMP conditions and detection

Real-Time LAMP was performed using thermal cycler CE-IVD M.C1000 (Bio-Rad, California, U.S.) with CFX96-IVD optical reaction module (Bio-Rad, California, U.S.) or QUANT Studio 7 Pro (ThermoFisher Scientific, Massachusetts, U.S.). The following reaction mixture was used:5.0 µL Universal WarmStart® LAMP 2X Master Mix (New England Biolabs, Massachusetts, U.S., catalogue number M1700), 0.5 µL EvaGreen (Biotium, California, U.S., catalogue number 31000), 0.15 µM F3, 0.15 µM B3, 1.20 µM FIP1, 1.20 µM FIP2, 1.20 µM FIP3, 1.20 µM BIP, 0.30 µM LF, 0.30 µM LB, 1.0 µM synthetic templates / DNase and RNase-free water ( no-template control (NTC)) / human DNA (wild-type).

The reaction volume was topped up to 10 µL with DNase and RNase-free water. The reaction was carried out for 50 min at 68 °C, with the fluorescence signal recorded every 60 s. The reaction temperature was selected based on obtaining the best results in the temperature gradient in the initial optimization stage of the method. Melting curve analysis was carried out after the amplification phase using the additional temperature profile of 65 °C–95 °C with increments of 0.5 °C, with each temperature level maintained for 5 s.

## Results

### Sensitivity and specificity of the method

The sensitivity of the method was determined by using a dilution series of synthetic templates modified to represent the human *EGFR* gene carrying the mutations present within exon 19:

- NM_005228.5(EGFR):c.2235_2249del (p.Glu746_Ala750del)
- NM_005228.5(EGFR):c.2240_2254del (p.Leu747_Thr751del)
- NM_005228.3:c.2239_2253del15
- NM_005228.3:c.2238_2252del15
- NM_005228.5(EGFR):c.2230_2249delinsGTCAA (p.Ile744_Ala750delinsValLys)

**Fig. 2:**
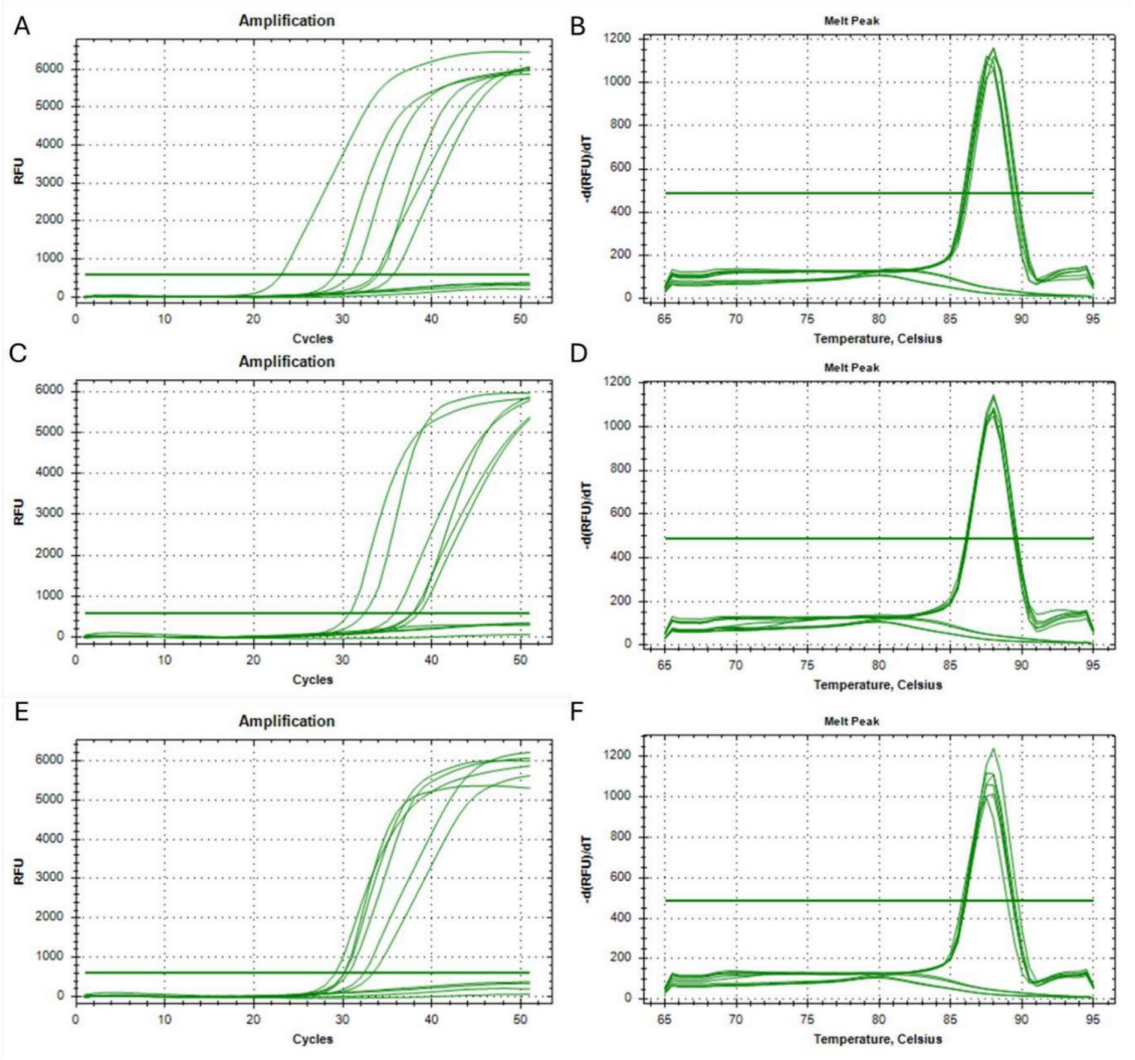
Amplification curves created during Real-Time LAMP (**A, C, E**) and the melting curves of the amplification products (**B, D, F**). Results represent dilution series of 250–1000 copies of modified synthetic templates representing the human EGFR gene carrying the mutations: NM_005228.5(EGFR):c.2235_2249del (p.Glu746_Ala750del) (A and B); NM_005228.5(EGFR):c.2240_2254del (p.Leu747_Thr751del) and NM_005228.3:c.2239_2253del15 and NM_005228.3:c.2238_2252del15 (C and D); NM_005228.5(EGFR):c.2230_2249delinsGTCAA (p.Ile744_Ala750delinsValLys) (E and F). Control samples containing human genetic material extracted from a healthy person (wild-type) or DNase and RNase-free water (NTC) were added to each analysis and are represented by amplification curves visible under the threshold line.

The limit of detection was evaluated within the rage of 250–1000 copies of the respective gBlock template per reaction. The amplification stage was performed by real-time fluorescence detection followed by melting-curve analysis where dissociation temperature readouts were within the range of 87.5–88.5 °C. The specificity was assessed using control samples containing 1) human genetic material extracted from a healthy person (wild-type) or 2) DNase and RNase-free water (NTC). The reaction for each type of sample was carried out in duplicate. The time required for the detection of the fluorescence signal emitted from individual samples is presented in Table 2, in addition Fig. 2 shows amplification curves created during reaction and melting curves of amplified product.

**Table 2:** The times required to obtain an exponential increase in the quantity of product in Real-Time LAMP reactions with synthetic templates at different concentrations (250–1000 copies) to evaluate the sensitivity of the method.

EGFR mutation variant	Sample type	Fluorescence baseline crossing time [min]
**NM_005228.5(EGFR):c.2235_2249del (p.Glu746_Ala750del)**	NTC	Undetermined
	NTC	Undetermined
	Human DNA (wild-type)	Undetermined
	Human DNA (wild-type)	Undetermined
	250 copies	31.01
	250 copies	33.58
	500 copies	34.03
	500 copies	35.64
	1000 copies	23.08
	1000 copies	29.13
**NM_005228.5(EGFR):c.2240_2254del (p.Leu747_Thr751del); NM_005228.3:c.2239_2253del15; NM_005228.3:c.2238_2252del15**	NTC	Undetermined
	NTC	Undetermined
	Human DNA (wild-type)	Undetermined
	Human DNA (wild-type)	Undetermined
	250 copies	35.84
	250 copies	37.77
	500 copies	37.93
	500 copies	38.51
	1000 copies	30.74
	1000 copies	32.41
**NM_005228.5(EGFR):c.2230_2249delinsGTCAA (p.Ile744_Ala750delinsValLys)**	NTC	Undetermined
	NTC	Undetermined
	Human DNA (wild-type)	Undetermined
	Human DNA (wild-type)	Undetermined
	250 copies	30.11
	250 copies	33.31
	500 copies	30.54
	500 copies	32.30
	1000 copies	29.10
	1000 copies	30.10

The results show that our method enables the detection of the NM_005228.5(EGFR):c.2235_2249del (p.Glu746_Ala750del) mutation, the NM_005228.5(EGFR):c.2240_2254del (p.Leu747_Thr751del) mutation, the NM_005228.3:c.2239_2253del15 mutation or the NM_005228.3:c.2238_2252del15 mutation, or the NM_005228.5(EGFR):c.2230_2249delinsGTCAA (p.Ile744_Ala750delinsValLys) mutation at a minimum quantity of 250 copies of target sequence.

The absence of an amplification product in the sample containing human genetic material extracted from a healthy person (wild-type) or DNase and RNase-free water (NTC) confirms the specificity of the primers to the genetic alteration in question. What is more, the specificity of the method was confirmed by the analysis of the melting curves (Fig. 1), which was the same for each product in dilution series.

### Role of FIP/BIP primer on the mechanism of LAMP amplification reaction

The effect of FIP and BIP primers on the mechanism of the LAMP reaction was analysed by carrying out reactions with and without FIP or BIP primers present. The purpose of this experiment was to determine whether a modification within the FIP or BIP primer would be crucial for the reaction mechanism, i.e. whether the binding or not of these primers to the targeted sequences determines the amplification. Reactions including gBlock® fragments representing a portion of the human *EGFR* gene with NM_005228.5(EGFR):c.2235_2249del (p.Glu746_Ala750del) mutation in exon 19 were performed in four replicates. Controls with DNase and RNase-free water (NTC) were run simultaneously and performed in duplicate. The time required to obtain an exponential increase in the product by Real-Time LAMP is presented in Table 3.

**Table 3:** The times required to obtain an exponential increase in the quantity of product in the Real-Time LAMP with or without FIP or BIP primers present.

Primers used in reaction mixtures	Fluorescence baseline crossing time [min]
FIP + BIP Repeat 1	32.11
FIP + BIP Repeat 2	27.54
FIP + BIP Repeat 3	26.20
FIP + BIP Repeat 4	30.01
NTC, FIP + BIP Repeat 1	Undetermined
NTC, FIP + BIP Repeat 2	Undetermined
Absence of FIP Repeat 1	Undetermined
Absence of FIP Repeat 2	Undetermined
Absence of FIP Repeat 3	Undetermined
Absence of FIP Repeat 4	Undetermined
NTC, Absence of FIP Repeat 1	Undetermined
NTC, Absence of FIP Repeat 2	Undetermined
Absence of BIP Repeat 1	Undetermined
Absence of BIP Repeat 2	Undetermined
Absence of BIP Repeat 3	Undetermined
Absence of BIP Repeat 4	Undetermined
NTC, Absence of BIP Repeat 1	Undetermined
NTC, Absence of BIP Repeat 2	Undetermined

**Fig. 3:**
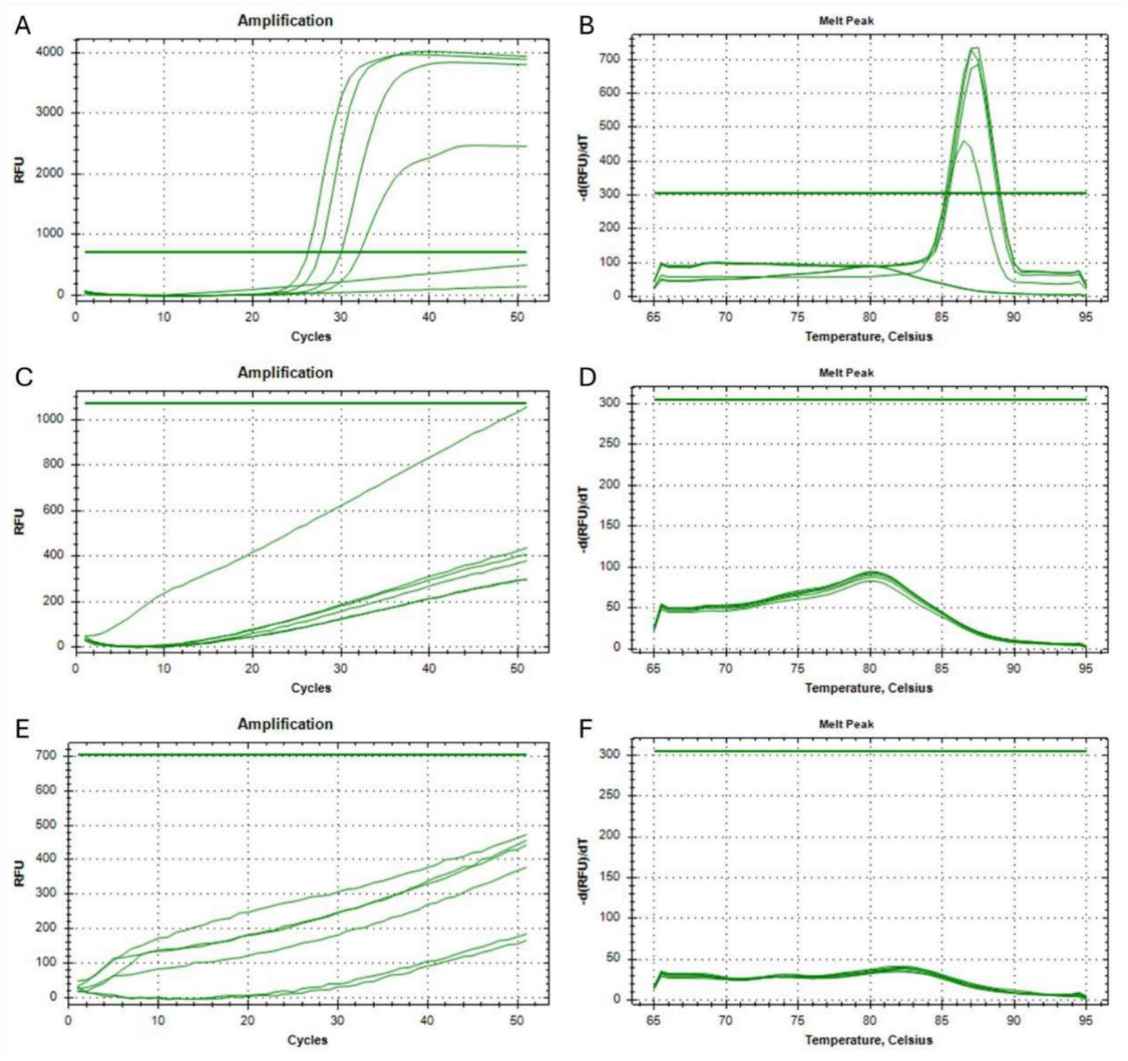
The amplification curves created during Real-Time LAMP (**A,C,E**) and melting curves of products (**B, D, F**). The reactions were carried out with all primers (**A,B**), without FIP (**C,D**), without BIP (**E,F**) present.

The amplification product was only observed in the presence of all primers in the reaction mixture (Fig. 3 A, B), while in the absence of the FIP primer (Fig. 3 C, D) or BIP primer (Fig. 3 E, F) the product was absent.

The results showed that both the FIP and the BIP primers play a crucial role in the LAMP reaction. At the same time, the mechanism of the LAMP reaction clearly indicates that the BIP primer is the functional equivalent of the FIP primer acting on the leading strand. Although our solution utilised the F2 primer design technique, the same approach can also be applied to the design of the B2 primer, thus allowing for the detection of a nucleotide variant using the leading strand and the BIP primer.

### Evaluation of primers modification capabilities

**Fig. 4:**
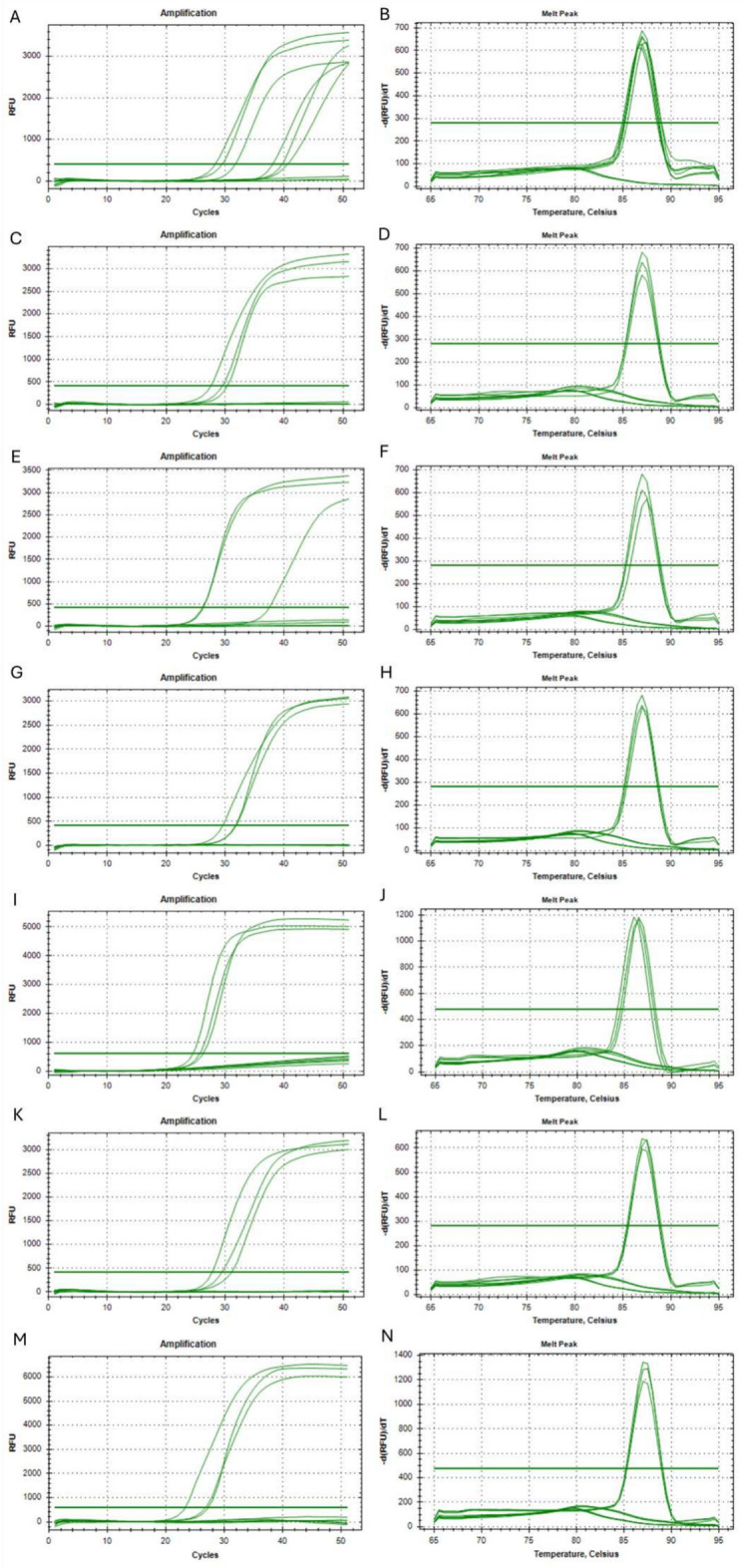
The amplification curve created during Real-Time LAMP reaction (**A, C, E, G, I, K, M**) and melting curves of the products (**B, D, F, H, J, L, N**). The reactions were carried out with primers with different modification lengths: 1 nucleotide (**A, B**), 2 nucleotides (**C, D**), 3 nucleotides (**E, F**), 4 nucleotides (**G, H**), 5 nucleotides (**I, J**), 6 nucleotides (**K, L**), and 7 nucleotides (**M, N**). Control samples containing human genetic material extracted from a healthy person (wild-type) or DNase and RNase-free water (NTC) were added to each analysis and are represented by amplification curves visible under the threshold line.

In order to determine the minimum and maximum number of nucleotides within the F2 primer, that must be identical to the nucleic acid sequence after the variant containing insertion and/or deletion, 7 different primers were designed. Each was modified within the 3’-end so that its final 1/2/3/4/5/6/7 nucleotides were identical to the nucleic acid sequence after the insertion and/or deletion of the genetic variant. The sample set for the analysis of each modified primers included a reaction with a 1000 copies of synthetic template modified to represent the human *EGFR* gene with the NM_005228.5(EGFR):c.2235_2249del (p.Glu746_Ala750del) mutation in exon 19 and also two controls, one with human genetic material extracted from a healthy person (wild-type) and the other with DNase and RNase-free water (NTC). The reaction for each sample type was carried out in triplicate. Table 4 shows the times required to obtain an exponential increase in the quantity of product in the Real-Time LAMP reaction. Where the primer had a mismatch related to only one nucleotide, the product of reaction was present both with a synthetic template and sample containing a human wild-type DNA, whereas no signal was obtained in NTC sample (Fig. 4 A, B). This result confirms that modification of the F2 primer within the range of a single nucleotide does not allow for a specific detection of the genetic variant. In the case of primers with 2 to 7 mismatched nucleotides the product of reaction was present only in samples containing a synthetic template with mutations to be detected, with no signal obtained in controls samples (wild-type and NTC, Fig. 4 C-N). These results confirm that modification of the F2 primer within the range of 2 to 7 nucleotides allows for specific detection of the genetic variant with a good discrimination between wild-type DNA and altered genetic sequence (insertion or deletion).

**Table 4:** The times required to obtain an exponential increase in the quantity of product in the Real-Time LAMP reactions with modified F2 primes.

F2 primer modification	Sample type	Fluorescence baseline crossing time [min]
**1 nucleotide long modification**	NTC	Undetermined
	NTC	Undetermined
	NTC	Undetermined
	Human DNA (wild-type)	31.76
	Human DNA (wild-type)	38.13
	Human DNA (wild-type)	40.67
	1000 copies of synthetic template	28.34
	1000 copies of synthetic template	29.51
	1000 copies of synthetic template	39.71
**2 nucleotide long modification**	NTC	Undetermined
	NTC	Undetermined
	NTC	Undetermined
	Human DNA (wild-type)	Undetermined
	Human DNA (wild-type)	Undetermined
	Human DNA (wild-type)	Undetermined
	1000 copies of synthetic template	27.57
	1000 copies of synthetic template	29.53
	1000 copies of synthetic template	30.37
**3 nucleotide long modification**	NTC	Undetermined
	NTC	Undetermined
	NTC	Undetermined
	Human DNA (wild-type)	Undetermined
	Human DNA (wild-type)	Undetermined
	Human DNA (wild-type)	Undetermined
	1000 copies of synthetic template	26.15
	1000 copies of synthetic template	26.25
	1000 copies of synthetic template	37.46
**4 nucleotide long modification**	NTC	Undetermined
	NTC	Undetermined
	NTC	Undetermined
	Human DNA (wild-type)	Undetermined
	Human DNA (wild-type)	Undetermined
	Human DNA (wild-type)	Undetermined
	1000 copies of synthetic template	29.60
	1000 copies of synthetic template	31.75
	1000 copies of synthetic template	31.90
**5 nucleotide long modification**	NTC	Undetermined
	NTC	Undetermined
	NTC	Undetermined
	Human DNA (wild-type)	Undetermined
	Human DNA (wild-type)	Undetermined
	Human DNA (wild-type)	Undetermined
	1000 copies of synthetic template	24.47
	1000 copies of synthetic template	25.63
	1000 copies of synthetic template	26.21
**6 nucleotide long modification**	NTC	Undetermined
	NTC	Undetermined
	NTC	Undetermined
	Human DNA (wild-type)	Undetermined
	Human DNA (wild-type)	Undetermined
	Human DNA (wild-type)	Undetermined
	1000 copies of synthetic template	27.80
	1000 copies of synthetic template	29.43
	1000 copies of synthetic template	31.10
**7 nucleotide long modification**	NTC	Undetermined
	NTC	Undetermined
	NTC	Undetermined
	Human DNA (wild-type)	Undetermined
	Human DNA (wild-type)	Undetermined
	Human DNA (wild-type)	Undetermined
	1000 copies of synthetic template	23.24
	1000 copies of synthetic template	26.75
	1000 copies of synthetic template	27.18

### Utilization of B2 primers’ modifications for mutation detection

**Fig. 5:**
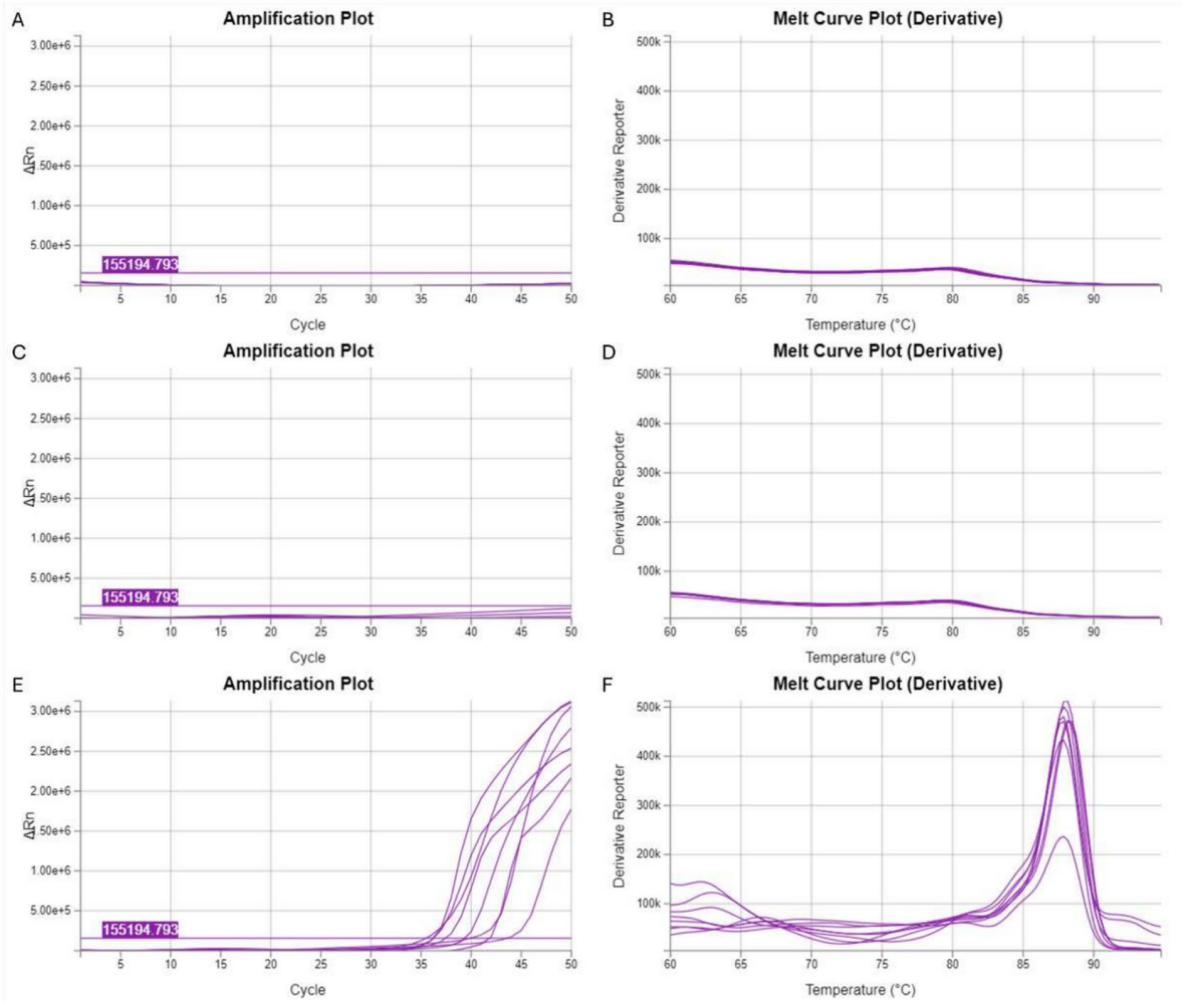
Amplification curves creating during Real-Time LAMP reaction (**A, C, E**) and melting curves of the products (**B, D, F**). The reactions were carried out with DNase and RNase-free water (NTC) (**A, B**), modified gBlock® representing fragment of the human *EGFR* gene with NM_005228.5(EGFR):c.2235_2249del (p.Glu746_Ala750del) (**C, D**) and unmodified gBlock® representing fragment of the human *EGFR* gene with NM_005228.5(EGFR):c.2235_2249del (p.Glu746_Ala750del) (**E, F**).

To verify whether our method of primer modification could also be applied to the B2 primer, we set up a reaction with specifically designed synthetic template i.e. gBlock® representing fragment of the human *EGFR* gene with NM_005228.5(EGFR):c.2235_2249del (p.Glu746_Ala750del) with 10 nucleotides inserted into the B2 primer attachment site. The reaction for each type of sample was carried out in eight replicates. Table 5 shows the time required to obtain an exponential increase in the quantity of product in the Real-Time LAMP reaction. We observed product in samples with gBlock® representing fragment of the human *EGFR* gene with NM_005228.5(EGFR):c.2235_2249del (p.Glu746_Ala750del) (Fig. 5 E, F), and no signal with the modified gBlock® representing fragment of the human *EGFR* gene with NM_005228.5(EGFR):c.2235_2249del (p.Glu746_Ala750del) with insertion of 10 nucleotides (Fig. 5. C, D) or the NTC samples (Fig. 5 A, B). The absence of product with modified gBlock® confirmed that like F2, B2 is sensitive to changes within the detected sequence. If we treat the modified gBlock® as wild-type and unmodified gBlock® as mutant with deletion of 10 nucleotides, the absence of product in reaction with wild-type and present of product with mutant proves that mutation would be detected. This result confirms that modification of the B2 primer can facilitate specific detection of the genetic variant.

**Table 5:** The times required to obtain an exponential increase in the quantity of product in the Real-Time LAMP reactions with modified gBlock® representing fragment of the human *EGFR* gene with NM_005228.5(EGFR):c.2235_2249del (p.Glu746_Ala750del), unmodified gBlock® representing fragment of the human *EGFR* gene with NM_005228.5(EGFR):c.2235_2249del (p.Glu746_Ala750del) and NTC.

Sample type	Fluorescence baseline crossing time [min]
NTC	Undetermined
NTC	Undetermined
NTC	Undetermined
NTC	Undetermined
NTC	Undetermined
NTC	Undetermined
NTC	Undetermined
NTC	Undetermined
1000 copies of modified synthetic template	Undetermined
1000 copies of modified synthetic template	Undetermined
1000 copies of modified synthetic template	Undetermined
1000 copies of modified synthetic template	Undetermined
1000 copies of modified synthetic template	Undetermined
1000 copies of modified synthetic template	Undetermined
1000 copies of modified synthetic template	Undetermined
1000 copies of modified synthetic template	Undetermined
1000 copies of synthetic template	35.54
1000 copies of synthetic template	36.27
1000 copies of synthetic template	36.28
1000 copies of synthetic template	37.54
1000 copies of synthetic template	39.38
1000 copies of synthetic template	40.00
1000 copies of synthetic template	41.81
1000 copies of synthetic template	43.78

### Specificity of primers in mixture of mutated and wild-type DNA

**Fig. 6:**
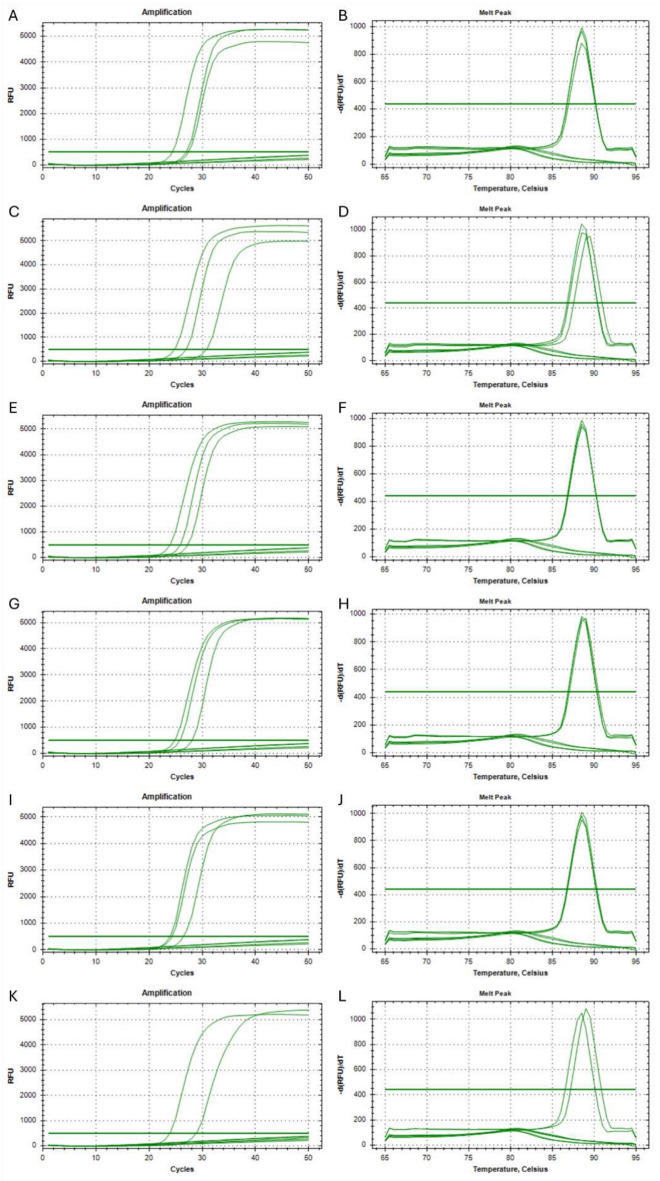
Amplification curve created during Real-Time LAMP reaction (**A, C, E, G, I, K**) and melting curves of the products (**B, D, F, H, J, L**). The reactions were carried out with gBlock® representing fragment of the human *EGFR* gene with NM_005228.5(EGFR):c.2235_2249del (p.Glu746_Ala750del) with VAF of 100% (**A, B**), with gBlock® representing fragment of the human *EGFR* gene with NM_005228.5(EGFR):c.2235_2249del (p.Glu746_Ala750del) and wild-type DNA in the same reaction with VAF of 5% (**C, D**), 1% (**E, F**), 0.5% (**G, H**), 0.1% (**I, J**), 0.01% (**K, L**). Control samples containing human genetic material extracted from a healthy person (wild-type) or DNase and RNase-free water (NTC) were added to each analysis and are represented by amplification curves visible under the threshold line.

Bearing in mind that oncological material usually contains a mixture of mutant and healthy cells, we assessed the specificity of the designed primers against the mutant DNA in the presence of wild-type DNA in the same reaction. We performed reaction in the presence of not only the gBlock® representing fragment of the human EGFR gene with NM_005228.5(EGFR):c.2235_2249del (p.Glu746_Ala750del), but also human genetic material extracted from a healthy person (wild-type). Moreover, we performed additional control reactions with 1) human genetic material extracted from a healthy person (wild-type), 2) DNase and RNase-free water (NTC). The reaction for each sample type was carried out in triplicate. The experiment was performed with a mixture of wild-type DNA and mutant DNA with a variant allele frequency (VAF) of 0.01%, 0.1%, 0.5%, 1%, 5% and with mutant DNA with VAF of 100%. The time required for the detection of the fluorescence signal from each individual samples is presented in Table 6. Figure 6 shows amplification curves created during reaction and melting curves of amplified product. We observed signal in experimental samples with VAF of 0.1%, 0.5%, 1%, 5% and 100% in all repetitions, for VAF of 0.01% we observed products in two out of three repetitions. A lack of signal was observed in samples with only wild-type DNA and NTC. The results confirm the specificity of designed primers to mutant DNA even in the presence of wild-type DNA with the lowest VAF, which was 0.1%.

**Table 6:** The time required to obtain an exponential increase in the quantity of product in the Real-Time LAMP reactions with gBlock® representing fragment of the human *EGFR* gene with NM_005228.5(EGFR):c.2235_2249del (p.Glu746_Ala750del) with VAF of 100%, with to obtain an exponential increase in the quantity of product in the Real-Time LAMP reactions with gBlock® representing fragment of the human *EGFR* gene with NM_005228.5(EGFR):c.2235_2249del (p.Glu746_Ala750del) and human DNA (wild-type) in a single reaction mixture with VAF of 5%, 1%, 0,5%, 0.1% and 0.01%, and controls (NTC; human DNA wild-type).

Sample type	Fluorescence baseline crossing time [min]
NTC	Undetermined
NTC	Undetermined
NTC	Undetermined
Human DNA (wild-type)	Undetermined
Human DNA (wild-type)	Undetermined
Human DNA (wild-type)	Undetermined
1000 copies of synthetic template VAF 100%	24.30
1000 copies of synthetic template VAF 100%	26.98
1000 copies of synthetic template VAF 100%	27.32
1000 copies of synthetic template and Human DNA (wild-type) VAF 5%	24.73
1000 copies of synthetic template and Human DNA (wild-type) VAF 5%	26.95
1000 copies of synthetic template and Human DNA (wild-type) VAF 5%	30.73
1000 copies of synthetic template and Human DNA (wild-type) VAF 1%	23.92
1000 copies of synthetic template and Human DNA (wild-type) VAF 1%	25.82
1000 copies of synthetic template and Human DNA (wild-type) VAF 1%	27.23
1000 copies of synthetic template and Human DNA (wild-type) VAF 0.5%	24.79
1000 copies of synthetic template and Human DNA (wild-type) VAF 0.5%	25.72
1000 copies of synthetic template and Human DNA (wild-type) VAF 0.5%	28.06
1000 copies of synthetic template and Human DNA (wild-type) VAF 0.1%	23.94
1000 copies of synthetic template and Human DNA (wild-type) VAF 0.1%	24.25
1000 copies of synthetic template and Human DNA (wild-type) VAF 0.1%	26.41
250 copies of synthetic template and Human DNA (wild-type) VAF 0.01%	23.85
250 copies of synthetic template and Human DNA (wild-type) VAF 0.01%	28.81
250 copies of synthetic template and Human DNA (wild-type) VAF 0.01%	Undetermined

## Discussion

The increasing dependence on the detection of genetic variants in precision medicine, including oncology, illustrates the need for rapid and reliable genetic testing. The lack of timely diagnosis is largely due to the sophisticated workflows of the diagnostic techniques currently utilised^25^. These techniques require state-of the art laboratory equipment, sophisticated software, and highly trained laboratory personnel, together with invasive sample collection methods based on tissue sampling. In addition, the multi-step nature of these methods makes it difficult to adapt to a fully automated system or rapid point-of-care testing (POCT) equipment. There are numerous advantages that make isothermal amplification methods, such as LAMP, a perfect diagnostic tool for the detection of genomic variation. LAMP is the optimal technique when fast and accurate diagnosis is required at a relatively low price thanks to its many unique features. The LAMP method is a single-step reaction, with short reaction times and low equipment requirements. Previous studies show that LAMP assays offer competitive pricing, around 43% less than those of PCR-based approach^26^. Considering fact that, loop-mediated-isothermal-amplification can utilise lower complexity devices for performing amplification and signal readout, in many instances leading to equipment-less process, entry barrier for introducing molecular testing might be vastly reduced^27–29^. By incorporating non-modified primers, further work on developed technology can allow for naked eye result detection e.g. by utilising colorimetric readout^30^. Due to the isothermal nature of the method, it can also be used in simplified POCT devices, which in the case of personalised medicine approach, would not only facilitate rapid detection at the point of need, especially in conditions that require urgent treatment with drugs heavily dependent on pharmacogenomic status of a patient^31,32^. With the increasingly growing portfolio of drugs that require companion diagnostics as well as epidemiological challenges requiring faster detection of pathogen spread it is of crucial importance to develop technologies that not only allow for discrimination of mutation profiles^33,34^. Considering the simplicity of our method, that does not require additional modification of oligonucleotides or multi-stage detection systems with possible further optimisations allowing for instrument-free operation, it paves the way for deployment in decentralised testing scheme. Despite the simplicity of the method, and the low equipment requirements, the diagnostic parameters of our method are comparable with those already existing on the market such as qPCR or NGS. An extremely valuable advantage of the LAMP method is its POCT capacity and availability to perform the reaction using relatively simple devices, what does not cause a deterioration in diagnostic parameters. We have shown that limit of detection measured as VAF (variant allele frequency) can be extremely low for del-ins mutations equaling 0.01% VAF, comparing to qPCR (1.0% of VAF) or NGS (2–5% of VAF depending on the source of data). Because LAMP reaction is relatively simple amplification technique, thus the time to results (TTR) is much shorter (> 45 min) compared to other amplification techniques, 90 min and 7000 min respectively for qPCR and NGS as shown in table 7^35^. What is more, LAMP method is highly resistant to inhibitors found in biological samples and able to withstand low quality nucleic acid template. This allows for the use of difficult biological material for LAMP analysis such as DNA/RNA extracted from liquid biopsy samples often used in cancer detection, prognosis, and treatment selection. The use of histopathological material collected during biopsy is not only more invasive for the patient, but the extraction of genetic material from such samples is more difficult, leading to extended analysis time and delays to further treatment^36–38^. Thanks to its high resistance to inhibitors, LAMP may be performed using a rapidly processed sample or even with crude biological samples simplifying and significantly speeding up the whole diagnostic process without negatively effecting sensitivity or specificity^13,39^. To enhance the study’s practical impact, it is crucial to evaluate the simplicity and cost-effectiveness of the technology, which allows for rapid and accurate diagnosis without the need for sophisticated equipment or highly trained personnel. Although, this study has already tested developed method with human genomic DNA from healthy donor, further validation studies using clinical samples with known mutation profile are planned to ensure the method’s reliability and accuracy in real-world settings. This involves testing the method with a variety of biological samples, including liquid biopsies, to confirm its effectiveness in detecting genetic variations. To offer full potential of POCT approach it is important to combine molecular biology techniques with recent developments in the lab-on-chip systems, together with mechanical and software design to offer versatile, fully autonomous diagnostic platform that will address needs of decentralised testing approach^40,41^ Table 7.

**Table 7:** Comparison between three common detection techniques dedicated for mutation detection, including findings related to described isothermal method (LAMP).

Method	POCT capacity	TTR [min]	LOD – VAF (Variant Allele Frequency)	Specificity	Type of sample
Genomtec LAMP technique	Yes	< 45 min	0.01% VAF	> 98%	Tumor biopsies, FFPE, cfDNA
qPCR	Moderate	> 90 min	1.0% VAF	> 98%	Tumor biopsies, FFPE, cfDNA
NGS	No	> 7000 min	2–5% VAF	> 98%	Tumor biopsies, FFPE

Considering the advantages of LAMP technology, we have created a new approach for the detection of genetic variations utilizing LAMP technology, which may be used for the point-of-care testing. The developed methodology consists of a modification of 2 to 7 nucleotides at the 3’-end of F2 primer or 2 to 7 nucleotides at the 3’-end of B2 primer, so that the sequence is identical or reverse and complimentary to nucleotide acid sequence after the targeted variant. At the same time the remaining portion of the primers are designed according to typical LAMP workflow. With this approach in the presence of wild-type nucleic acid template, the 3’-ends of F2 or B2 primers remain separated from the template preventing amplification. Contrary to other isothermal amplification methods dedicated to oncogenic variant detection, our approach does not utilize fluorescent-label probes that would otherwise increase the costs and make the diagnostic process more complex, while increasing the chance of false positive results^42^. Our approach utilizes florescence signal generated by intercalating dye in the form of EvaGreen dye, which is inexpensive and has universal applications. Furthermore, our approach also enables the diagnosis of deletion/insertion events that affect a relatively small region, making the detection of even single nucleotide displacements achievable using this method. Our approach allows for simplified multiplex detection of genetic variants without the need of increasing the complexity of each assay by incorporating probe-based detection, while maintaining specificity by allowing amplification only when the targeted variant is present. Our approach can be used not only in oncology, but any sector of diagnostics where a need is to detect genetic variations i.e. insertions or/and deletions in biological material.

The process of developing this new methodological approach included a series of analyses. First, we evaluated how the course of the amplification reaction depends on the presence of the FIP or BIP primer in the reaction mixture. Our analyses unequivocally confirmed that both the FIP and the BIP primers play a crucial role in the course of the LAMP reaction. At the same time, the results clearly indicate that the BIP primer is the functional equivalent of the FIP primer although acting on the leading strand. The F2 primer design technique we have developed can also be applied to the design of the B2 primer, thus allowing for the detection of a nucleotide variant using the leading strand along with the BIP primer. This assumption was further confirmed by designing a reaction with specifically prepared synthetic template i.e. gBlock® representing a fragment of the human *EGFR* gene with NM_005228.5(EGFR):c.2235_2249del (p.Glu746_Ala750del), which was reverse and complementary transcribed utilising the 10 additional nucleotides inserted into the B2 primer annealing site. The absence of product with the modified gBlock® confirmed that B2, like F2, is sensitive to changes within the detected sequence. Analysis of the specificity and sensitivity of the method confirmed that the assay we have developed provides a diagnostic solution demonstrating adequate specificity against the expected product, and a sensitivity estimated at 250 copies of the target sequence.

## Conclusion

The LAMP technique has the potential to be successfully utilised for detection of several types of genetic variants, proven both by Genomtec and numerous other research groups. Due to its simple, fast, and low-cost diagnostic process, which does not require any sophisticated equipment or highly qualified personnel, the assay design approach developed by Genomtec is the perfect solution for point-of-care variant detection. Moreover, thanks to its ability to use a wide variety of biological samples, including liquid biopsy, minimally pre-processed or crude biological sample, the use of LAMP-based approach for point-of-care testing is both easy and convenient^39,43^.

## Supplementary Information

Supplementary Information.

## Data Availability

The datasets analysed during the current study are included in this published article and its supplementary information or available in the ClinVar repository under: http://www.ncbi.nlm.nih.gov/clinvar/RCV000150617.4http://www.ncbi.nlm.nih.gov/clinvar/RCV000154236.4http://www.ncbi.nlm.nih.gov/clinvar/RCV000150616.4
