# Supplementary material for: A new approach for the detection of genetic alterations utilizing modified loop-mediated isothermal amplification reaction (LAMP)
Source: Sci Rep. 2025 Mar 8;15:8071. doi: 10.1038/s41598-025-93086-2 (PMC11889157; doi:10.1038/s41598-025-93086-2)
Supplement: Supplementary file 1 — Supplementary Information. [file 41598_2025_93086_MOESM1_ESM.pdf]

gBlock 1 representing NM_005228.5(EGFR):c.2235_2249del (p.Glu746_Ala750del):

GCATTTTTATGAAAGGGGCCATTGACCTTGCCATGGGGTGCAGCACAGGGCGGGAGGAG
GGCCGCCTCTCACCGCACGGCATCAGAATGCAGCCCAGCTGAAATGGGCTCATCTTCGT
TTGCTTCTTCTAGATCCTCTTTGCATGAAATCTGATTTCAGTTAGGCCTAGACGCAGCATCAT
TAAATTCTGGATGAAATGATCCACACGGACTTTATAACAGGCTTTACAAGCTTGAGATTCTTTT
ATCTAAATAATCAGTGTGATTCGTGGAGCCCAACAGCTGCAGGGCTGCGGGGGCGTCACA
GCCCCCAGCAATATCAGCCTTAGGTGCGGCTCCACAGCCCCAGTGTCCCTCACCTTCGG
GGTGCATCGCTGGTAACATCCACCCAGATCACTGGGCAGCATGTGGCACCATCTCACAAT
TGCCAGTTAACGTCTTCCTTCTCTCTCTGTCATAGGGACTCTGGATCCCAGAAGGTGAGAA
GTTAAAATTCCCGTCGCTATCAAAACATCTCCGAAAGCCAACAAGGAAATCCTCGATGTGA
GTTTCTGCTTTGCTGTGTGGGGGTCCATGGCTCTGAACCTCAGGCCCACCTTTTCTCATGT
CTGGCAGCTGCTCTGCTCTAGACCCTGCTCATCTCCACATCCTAAATGTTCACTTTCTATGT
CTTTCCCTTTCTAGCTCTAGTGGGTATAACTCCCTCCCCTTAGAGACAGCACTGGCCTCTC
CCATGCTGGTATCCACCCCAAAAGGCTGGAAACAGGCAATTACTGGCATCTACCCAGCAC
TAGTTTCTTGACACGCATGACGAGTGAGTGCTCTTGGTGAGCCTGGAGCATGGGTATTGTTT
TTGGTATTTTTTGGATGAAGAAATGGAGGCATAAAGAAATTG

gBlock 2 representing NM_005228.5(EGFR):c.2240_2254del (p.Leu747_Thr751del) or NM_005228.3:c.2239_2253del15 or NM_005228.3:c.2238_2252del15:

GCATTTTTATGAAAGGGGCCATTGACCTTGCCATGGGGTGCAGCACAGGGCGGGAGGAG
GGCCGCCTCTCACCGCACGGCATCAGAATGCAGCCCAGCTGAAATGGGCTCATCTTCGT
TTGCTTCTTCTAGATCCTCTTTGCATGAAATCTGATTTCAGTTAGGCCTAGACGCAGCATCAT
TAAATTCTGGATGAAATGATCCACACGGACTTTATAACAGGCTTTACAAGCTTGAGATTCTTTT
ATCTAAATAATCAGTGTGATTCGTGGAGCCCAACAGCTGCAGGGCTGCGGGGGCGTCACA
GCCCCCAGCAATATCAGCCTTAGGTGCGGCTCCACAGCCCCAGTGTCCCTCACCTTCGG
GGTGCATCGCTGGTAACATCCACCCAGATCACTGGGCAGCATGTGGCACCATCTCACAAT
TGCCAGTTAACGTCTTCCTTCTCTCTCTGTCATAGGGACTCTGGATCCCAGAAGGTGAGAA
AGTTAAAATTCCCGTCGCTATCAAGGAATCTCCGAAAGCCAACAAGGAAATCCTCGATGTG
AGTTTCTGCTTTGCTGTGTGGGGGTCCATGGCTCTGAACCTCAGGCCCACCTTTTCTCATGT
CTGGCAGCTGCTCTGCTCTAGACCCTGCTCATCTCCACATCCTAAATGTTCACTTTCTATGT
CTTTCCCTTTCTAGCTCTAGTGGGTATAACTCCCTCCCCTTAGAGACAGCACTGGCCTCTC
CCATGCTGGTATCCACCCCAAAAGGCTGGAAACAGGCAATTACTGGCATCTACCCAGCAC
TAGTTTCTTGACACGCATGACGAGTGAGTGCTCTTGGTGAGCCTGGAGCATGGGTATTGTTT
TTGGTATTTTTTGGATGAAGAAATGGAGGCATAAAGAAATTG

gBlock 3 representing NM_005228.5(EGFR):c.2230_2249delinsGTCAA (p.Ile744_Ala750delinsValLys):

GCATTTTTATGAAAGGGGCCATTGACCTTGCCATGGGGTGCAGCACAGGGCGGGAGGAG
GGCCGCCTCTCACCGCACGGCATCAGAATGCAGCCCAGCTGAAATGGGCTCATCTTCGT
TTGCTTCTTCTAGATCCTCTTTGCATGAAATCTGATTTCAGTTAGGCCTAGACGCAGCATCAT
TAAATTCTGGATGAAATGATCCACACGGACTTTATAACAGGCTTTACAAGCTTGAGATTCTTTT
ATCTAAATAATCAGTGTGATTCGTGGAGCCCAACAGCTGCAGGGCTGCGGGGGCGTCACA
GCCCCCAGCAATATCAGCCTTAGGTGCGGCTCCACAGCCCCAGTGTCCCTCACCTTCGG
GGTGCATCGCTGGTAACATCCACCCAGATCACTGGGCAGCATGTGGCACCATCTCACAAT

TGCCAGTTAACGTCTTCCTTCTCTCTCTGTCATAGGGACTCTGGATCCCAGAAGGTGAGAA
GTTAAAATTCCCGTCGCTGTCAAAACATCTCCGAAAGCCAACAAGGAAATCCTCGATGTGA
GTTTCTGCTTTGCTGTGTGGGGGTCCATGGCTCTGAACCTCAGGCCCACCTTTTCTCATGT
CTGGCAGCTGCTCTGCTCTAGACCCTGCTCATCTCCACATCCTAAATGTTCACTTTCTATGT
CTTTCCCTTTCTAGCTCTAGTGGGTATAACTCCCTCCCCTTAGAGACAGCACTGGCCTCTC
CCATGCTGGTATCCACCCCAAAAGGCTGGAAACAGGCAATTACTGGCATCTACCCAGCAC
TAGTTTCTTGACACGCATGACGAGTGAGTGCTCTTGGTGAGCCTGGAGCATGGGTATTGTTT
TTGGTATTTTTTGGATGAAGAAATGGAGGCATAAAGAAATTG

Modified gBlock 1:

GCATTTTTATGAAAGGGGCCATTGACCTTGCCATGGGGTGCAGCACAGGGCGGGAGGAG
GGCCGCCTCTCACCGCACGGCATCAGAATGCAGCCCAGCTGAAATGGGCTCATCTTCGT
TTGCTTCTTCTAGATCCTCTTTGCATGAAATCTGATTTCAGTTAGGCCTAGACGCAGCATCAT
TAAATTCTGGATGAAATGATCCACACGGACTTTATAACAGGCTTTACAAGCTTGAGATTCTTTT
ATCTAAATAATCAGTGTGATTCGTGGAGCCCAACAGCTGCAGGGCTGCGGGGGCGTCACA
GCCCCCAGCAATATCAGCCTTAGGTGCGGCTCCACAGCCCCAGTGTCCCTCACCTTCGG
GGTGCATCGCTGGTAACATCCACCCAGATCACTGGGCAGCATGTGGCACCATCTCACAAT
TGCCAGTTAACGTCTTCCTTCTCTCTCTGTCATAGGGACTCTGGATCCCAGAAGGTGAGAA
AGTTAAAATTCCCGTCGCTATCAAAACATCTCCGAAAGCCAACAAGGAAATCCTCGATGTG
AGTTTCTGCTTTGCTGTGTGGGGGTCCATGGCTCTGAACCTCAGGCCCACCTTTTCTCATGT
CTGGCAGCTGCTCTGCTCTAGACCCTGCTCATCTCCACATCCTAAATGTTCACTTTCTATGT
CTTTCCCTTTCTAGCTCTAGTGGGTATAACTCCCTCCCCTTAGAGACA**CTGTCATCTC**GCA
CTGGCCTCTCCCATGCTGGTATCCACCCCAAAAGGCTGGAAACAGGCAATTACTGGCATC
TACCCAGCACTAGTTTCTTGACACGCATGACGAGTGAGTGCTCTTGGTGAGCCTGGAGCAT
GGGTATTGTTTTTGGTATTTTTTGGATGAAGAAATGGAGGCATAAAGAAATTG
